# Simultaneous surgical treatment of liver metastases from colorectal cancer in the elderly through Habib's technique

**DOI:** 10.1186/1471-2482-13-S1-A49

**Published:** 2013-09-16

**Authors:** Xheseda Dumani, Gennaro Rizzo, Sebastiano Grassia, Vincenzo Tammaro, Nicola Carlomagno, Andrea Renda

**Affiliations:** 1 U.O.C. Chirurgia Generale ad Indirizzo Addominale, Università Federico II – via Pansini 5 Napoli, Italy

## Introduction

The liver is the most common site of metastasis from colo-rectal cancer (CRC) that may already be present up to 30% of cases the diagnosis of the primary tumor. In recent years, thanks to the improvement of surgical techniques and chemotherapy, the prognosis is improved and the criteria for a correct indication for surgery are less restrictive than in the past [1][2]. The behaviour in the presence of synchronous liver metastases (ME), however, is still controversial, and removal of the primary tumor and ME can occur at different times. In view of the improvement of surgical techniques and perioperative management of technological development, in some selected cases a contemporary approach can be planned. We have thus decided to conduct a retrospective study to evaluate whether there is increased risk of morbidity 'and mortality, as well as a significant increase in operative time, especially focusing on the influence of age in the simultaneous treatment of the primary tumor and ME.

## Methods

From 2004 to 2012, we treated 36 patients (23 males and 13 females) affected by CRC with liver metastases at diagnosis. We divided the patients into two groups:

Group A (21 patients) aged less than 70 y.o. (range 48-70) - M: F = 1:2

Group B (15 patients) > 70 years y.o. (range 71 - 84 years) - M: F = 1:3

Simultaneous treatment was based on the following inclusion criteria: patient's general health status, ASA score, characteristics of liver metastases (site, size and number) and percentage of healthy liver 3. Pre-operative investigations were blood tests, liver function test, colonoscopy, US abdomen, abdomen and pelvis CT scan or total body CT/PET, MRI, contrast-enhanced liver US and rectal endoscopic ultrasonography (for rectal cancer).

The patients were all operated on colectomy and liver metastases resection. Metastasectomies were always performed through Habib's technique which includes the activation of a bipolar device using radiofrequency 4.

Intra-operative blood loss, operative time, postoperative course, morbidity and mortality rates, hospitalization time were analysed and compared between two groups.

## Results

We performed 51 metastasectomies (in 33 patients) and 3 wedge resections. We never used Pringle’s manoeuvre. Mean intraoperative blood loss was 150 ml (range 100-350 cc) and only one patient required a blood transfusion. The mean increase of operation time was 35 minutes. Postoperative outcome was acceptable in all cases. Bowel motility started at 48-72 h. Mean drainage output was 180 cc at 24h and 70cc at 48h. Modest and transient increase in liver function tests verified in almost all cases. There were no major systemic complications, just one patient postoperatively suffered hepato-renal failure treated in intensive care unit. Patients were discharged on the 9th-12th day. There was no mortality. Results according to the different ages are reported in table 1.

**Table 1:** Results according to the different ages

	Intra-operative blood loss (cc)	Increase of operative time (minutes)	Morbidity (n)	Mortality (n)	Mean hospitalization time (days)
Group A	120 ml (range 80-320 cc)	35	2 (9,5%)	-	11

Group B	160 ml (range 100-350 cc)	35	3 (20%)	-	12

## Conclusions

Habib’s device allowed us to perform metastasecomies at the same time of colectomy without Pringle’s manoeuvre, with minimal blood loss and without excessive prolongation of the surgical time 5. In both groups postoperative haemorrhage and biliary leak were minimal, supporting the effectiveness of the technique. Our experience showed that Habib’s procedure is easy and safe 6. It is our opinion that such device allows liver resections to be carried out with minimal blood loss and low mortality and morbidity rates, and results are similar between young and old patients.

## References

[B1] Weber Jean-Christophe Navarra Giuseppe Jiao Long R Nicholls Joanna P Jensen Steen Lindkaer Habib Nagy A New Technique for Liver Resection Using Heat Coagulative Necrosis Annals of Surgery 2002 13 5 560 563 10.1097/00000658-200211000-00004 12409660 PMC1422612

[B2] Weber JC Navarra G Habib NA Bachellier P Jaeck D Laparoscopic radiofrequency–assisted liver resection Surg Endosc 2003 13 831 834 10.1007/s00464-002-4537-2 15768457

[B3] Rispoli C Rocco N Iannone L Amato B Developing guidelines in geriatric surgery: role of the grade system BMC Geriatrics 2009 13 SUPPL.1 A99

[B4] Ayav Ahmet Navarra Giuseppe Habib Nagy A Jiao Long R New Technique for Liver Resection Using Heat Coagulative Necrosis Annals of Surgery 2005 13 5 10.1097/00000658-200511000-00025 PMC1409856 16244556

[B5] Stavrou GA Tzias Z von Falck C Habib N Oldhafer KJ Hepatic resection using heat coagulative necrosis. First report of successful trisegmentectomy after hypertrophy induction Langenbecks Arch Surg 2007 13 1 95 97 10.1007/s00423-006-0118-5 17131152

[B6] Mastromarino R D’Angelo S Saglioccolo A Rizzo G Tammaro V Carlomagno N Renda A Preliminary results with habib’s procedure BMC Geriatrics 2011 13 Suppl 1 A32 10.1186/1471-2318-11-S1-A32

